# White matter hyperintensity on MRI and plasma Aβ42/40 ratio additively increase the risk of cognitive impairment in hypertensive adults

**DOI:** 10.1002/alz.14126

**Published:** 2024-09-04

**Authors:** Adam de Havenon, Rebecca F. Gottesman, Jeff D. Willamson, Natalia Rost, Richa Sharma, Vivian Li, Lauren Littig, Eric Stulberg, Guido J. Falcone, Shyam Prabhakaran, Andrea L. C. Schneider, Kevin N. Sheth, Nicholas M. Pajewski, Adam M. Brickman

**Affiliations:** ^1^ Department of Neurology Center for Brain and Mind Health Yale University School of Medicine New Haven Connecticut USA; ^2^ National Institute of Neurological Disorders and Stroke Bethesda Maryland USA; ^3^ Department of Internal Medicine Wake Forrest University School of Medicine Winston‐Salem North Carolina USA; ^4^ Department of Neurology Massachusetts General Hospital Boston Massachusetts USA; ^5^ Department of Neurology University of Utah Salt Lake City Utah USA; ^6^ Department of Neurology University of Chicago Chicago Illinois USA; ^7^ Department of Neurology Department of Biostatistics Epidemiology, and Informatics University of Pennsylvania Perelman School of Medicine Philadelphia Pennsylvania USA; ^8^ Department of Biostatistics and Data Science Wake Forrest University School of Medicine Winston‐Salem North Carolina USA; ^9^ Taub Institute for Research on Alzheimer's Disease and the Aging Brain and the Department of Neurology Columbia University New York New York USA

**Keywords:** Alzheimer's disease biomarkers, Aβ42/40 ratio, cerebrovascular pathology, cognitive impairment risk, dementia, vascular and amyloid pathways, white matter hyperintensity (WMH)

## Abstract

**INTRODUCTION:**

Dementia often involves comorbid Alzheimer's and vascular pathology, but their combined impact warrants additional study.

**METHODS:**

We analyzed the Systolic Blood Pressure Intervention Trial and categorized white matter hyperintensity (WMH) volume into highest versus lowest/mid tertile and the amyloid beta (Aβ)42/40 ratio into lowest versus mid/highest ratio tertile. Using these binary variables, we created four exposure categories: (1) combined low risk, (2) Aβ risk, (3) WMH risk, and (4) combined high risk.

**RESULTS:**

In the cohort of 467 participants (mean age 69.7 ± 7.1, 41.8% female, 31.9% nonwhite or Hispanic) during 4.8 years of follow‐up and across the four exposure categories the rates of cognitive impairment were 5.3%, 7.8%, 11.8%, and 22.6%. Compared to the combined low‐risk category, the adjusted hazard ratio for cognitive impairment was 4.12 (95% confidence interval, 1.71 to 9.94) in the combined high‐risk category.

**DISCUSSION:**

This study emphasizes the potential impact of therapeutic approaches to dementia prevention that target both vascular and amyloid pathology.

**Highlights:**

- White matter hyperintensity (WMH) and plasma amyloid (Aβ42/40) are additive risk factors for the development of cognitive impairment in the SPRINT MIND trial.
- Individuals in the high‐risk categories of both WMH and Aβ42/40 had a near fivefold increase in risk of cognitive impairment during 4.8 years of follow‐up on average.
- These findings suggest that treatment strategies targeting both vascular health and amyloid burden warrant further research.

## 1 BACKGROUND

Brain health is a leading concern of healthy aging, with cognitive decline presenting a particularly troubling challenge.^1^, ^2^, ^3^ While disease‐specific models often focus on cerebrovascular or Alzheimer's disease (AD) pathology in isolation, evidence suggests that the majority of dementia results from a combination of both.^4^, ^5^, ^6^, ^7^, ^8^ The least invasive measurement of AD amyloid beta (Aβ) pathology is plasma biomarkers. The ratio of plasma peptides Aβ42 to Aβ40 (Aβ42/40) is inversely correlated with the brain's burden of Aβ on positron emission tomography (PET) and the future development of AD.^9^, ^10^, ^11^ In contrast, the least invasive measurement of cerebrovascular pathology is brain pathology identified by magnetic resonance imaging (MRI). White matter hyperintensity (WMH) on MRI is a manifestation of chronic microvascular disease, typically caused by poor control of vascular risk factors such as hypertension.^12^, ^13^, ^14^, ^15^ According to a recent meta‐analysis, WMH is the best MRI biomarker of vascular cognitive impairment and dementia potentially because it results in more diffuse brain injury than other vascular biomarkers such as lacunes or cerebral microbleeds, which are typically localized.^16^, ^17^, ^18^, ^19^, ^20^

Although some publications suggest that Aβ pathology may result in WMH,^21^, ^22^ the association between Aβ and vascular pathology remains controversial.^23^ An alternative explanation is that WMH results from poor control of vascular risk factors and is independent from Aβ pathology.^24^, ^25^, ^26^ Prior analyses predicting the development of cognitive impairment have shown an additive relationship between vascular risk and Aβ burden measured on PET.^27^, ^28^, ^29^ However, this additive relationship has not been studied in a racially diverse cohort. In the current study, we perform a post hoc hypothesis‐generating analysis to investigate the potentially additive effect of WMH and the Aβ42/40 ratio on the risk of cognitive impairment in a diverse hypertensive population.

## 2 METHODS

### 2.1 Cohort

We performed a post hoc analysis of the Systolic Blood Pressure Intervention Trial (SPRINT).^30^ Originally initiated in 2010, SPRINT was a randomized clinical trial focused on hypertension treatment in individuals aged 50 years or older, which was terminated early in 2015 due to a favorable effect on the primary outcome (cardiovascular morbidity and mortality) and all‐cause mortality. SPRINT tested an intensive versus standard blood pressure goal (<120 vs <140 mm Hg). Our analysis used de‐identified SPRINT data, sourced from the National Heart, Lung, and Blood Institute (NHLBI) Biologic Specimen and Data Repository Information Coordinating Center (BioLINCC), which are publicly available.^31^ We obtained a local Institutional Review Board waiver for the de‐identified data. We included SPRINT participants who were enrolled in a substudy that obtained a baseline MRI and those who had a measurement of plasma AD biomarkers as part of the main trial, but only subjects aged ≥60 were eligible for the plasma AD biomarker trial.^32^

### 2.2 Exposure and outcome

The study exposures are WMH on MRI and the plasma Aβ42/40 ratio, both ascertained at baseline. Although neither biomarker is a perfect surrogate, in this analysis we treat WMH as a surrogate for vascular contributions to cognitive impairment and the Aβ42/40 ratio as a surrogate of cerebral amyloid burden. WMH volume was measured on three Tesla MRIs using a validated and standardized automated segmentation methodology previously described in this cohort.^33^ The Aβ42/40 ratio was measured in a plasma sample obtained at baseline. The sample was prepared at the study site according to protocol and then shipped frozen to a core laboratory. The measurement was then performed on a single molecule‐array (Simoa) HD‐1 analyzer platform using the Simoa Human Neurology 3‐Plex A assay. All samples were assayed in duplicate and were run with kits from the same lot for each analyte.^34^

The study outcome is cognitive decline, which was a SPRINT MIND secondary outcome and is the composite of probable dementia and mild cognitive impairment (MCI).^32^ As a secondary outcome, we focused on probable dementia (“dementia”) only but are inherently underpowered due to a low outcome rate. Cognitive status assessment in SPRINT involved a three‐step process. Participants underwent cognitive screening assessments at baseline, year 2, year 4, and at study closeout if it was more than 1 year from year 4. Participants who scored below study‐defined thresholds for the Montreal Cognitive Assessment (MoCA) or Functional Activities Questionnaire underwent further testing with an extended neuropsychological battery. The extended battery included tests reflecting memory (Hopkins Verbal Learning Test‐Revised delayed recall, the Modified Rey‐Osterrieth Complex Figure immediate recall, and Logical Memory I and II), processing speed (Trail Making Test Parts A and B and Digit Symbol Coding), executive function (Trail Making Test–Part B minus Part A and Digit Span), language (Boston Naming Test‐15 and Category Fluency–Animals), and global cognitive function (all tests included in the above domain scores).^30^ In addition to cognitive tests, participants’ functional status, depressive symptoms, and health status were assessed. A centralized expert adjudication panel independently reviewed and classified participants as having no cognitive impairment, MCI, or probable dementia based on standardized diagnostic criteria,^30^ with disagreements resolved by majority vote.

**RESEARCH IN CONTEXT**

1. **Systematic review**: We reviewed literature on dementia, focusing on cerebrovascular risk factors and Alzheimer's pathology. Afterwards, we proceeded with analyzing the Systolic Blood Pressure Intervention Trial data for the impact of white matter hyperintensity (WMH) and the plasma amyloid beta (Aβ)42/40 ratio on risk of cognitive impairment.
2. **Interpretation**: Our study underscores the significant role of vascular and amyloid biomarkers in increasing cognitive impairment risk. By categorizing risk based on WMH and Aβ42/40 levels, we found that the highest risk category of both biomarkers was associated with a nearly fivefold increase in the risk of developing cognitive impairment.
3. **Future directions**: Research should explore longitudinal relationships between these biomarkers and cognitive decline. Investigating intervention strategies that target both vascular and amyloid pathways in hypertensive adults and understanding the underlying interactions between these pathways will be crucial for advancing dementia treatment and prevention.

### 2.3 Statistical analysis

**FIGURE 1 alz14126-fig-0001:**
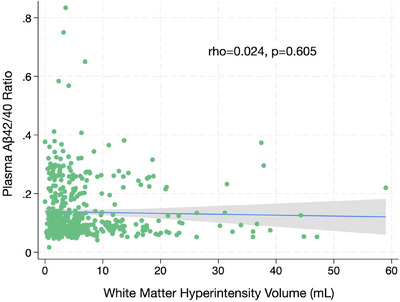
Scatter plot and linear fit with 95% confidence interval between white matter hyperintensity in mL and plasma amyloid beta (Aβ)42/40 ratio.

Both WMH volume and the Aβ42/40 ratio had a right skew (Figure 1), so we categorized them into tertiles to avoid problems with model specification. Although there are theoretic cut points for WMH and Aβ42/40,^19^, ^35^, ^36^ the measurement of both has inherent imprecision between different methodologies, making a tertile approach more generalizable. The relationship between the tertiles was visualized in a Sankey plot. We hypothesized that the top tertile of WMH (largest WMH burden) and the lowest tertile of the Aβ42/40 ratio (increased risk of Aβ burden) would be associated with the highest risk of developing cognitive impairment. To test the hypothesis, we first categorized WMH volume and Aβ42/40 into binary variables: highest tertile of WMH versus lowest/mid tertile of WMH and lowest tertile of the Aβ42/40 ratio versus the mid/highest Aβ42/40 ratio. We then created an ordinal four‐level exposure from these two binary variables: (1) combined low risk (lowest/mid tertiles of WMH, mid/highest tertiles of the Aβ42/40 ratio), (2) Aβ risk (lowest/mid tertiles of WMH, lowest tertile of the Aβ42/40 ratio), (3) WMH risk (highest tertile of WMH, mid/highest tertiles of the Aβ42/40 ratio), and (4) combined high risk (highest tertile of WMH, lowest tertile of Aβ42/40 ratio).[Fig alz14126-fig-0001]

The primary analysis is a time‐to‐event Cox regression. We report unadjusted and adjusted models. The adjusted model fit to the primary outcome included baseline age in quintiles, sex (male, female), self‐reported race/ethnicity (white, black, Hispanic, other), education (<college, college, graduate school), physical activity level (inactive, active 1 to 4 days/week, active ≥5 days/week, defined by self‐report as “activities that make you sweat, increase your heart rate or increase your breathing”), alcohol use (none, ≥1 drink/day), self‐reported depression, and SPRINT randomization arm. The adjusted model for the secondary outcome of probable dementia only included age, sex, and race/ethnicity due to the risk of overfitting. For all Cox models, we visually evaluated the proportional hazards assumption using the Schoenfeld residuals.^37^

Although there were no outcome events until the first cognitive adjudication visit 2 years from enrollment, we did not change the index date to the year 2 visit. This was done to mirror the approach that was used in the SPRINT MIND study.^30^ However, to exclude bias from interval censoring, we also performed the analysis with the index time set at 1.9 years from enrollment and the results were consistent, thus excluding that bias. We created a Kaplan–Meier curve for the cumulative incidence of cognitive impairment by exposure status and for that figure, to assist in visual interpretation, set the index at 1.9 years. This results in the exclusion of one individual who died at 1.2 years of follow‐up.

To determine if the exposures were multiplicative, additive, or superadditive, we added the interaction term of binary WMH*Aβ42/40 to the adjusted Cox model fit to the primary outcome and also derived the relative excess risk due to interaction (RERI), attributable proportion, and synergy index.^38^, ^39^, ^40^ The RERI assess two‐way interactions in an additive model of relative risk. Attributable proportion reflects the extra risk from combined exposures A and B beyond their additive risks. The synergy index turns the RERI into a ratio for a comparative view of risk interplay.^41^

In the adjusted model from the primary analysis, we tested the interaction between the exposure categories and binary covariates: age (<70 vs ≥70), sex (male vs female), and race/ethnicity (non‐Hispanic white vs nonwhite so that there are enough events). We a priori did not test for an interaction with the treatment arm because the potential for spurious findings related to treatment could have unintended consequences. We also performed an exploratory analysis where we included WMH and Aβ42/40 as continuous variables, but to address their right skew we cube‐root transformed them. We used the interaction of these variables and marginal effects of their centered mean ± two standard deviations to create a 3D plot of the hazard for the primary outcome.

We performed three sensitivity analyses, all in adjusted models from the primary analysis. The first accounted for all‐cause death as a competing event in a Fine‐Gray model. The second standardized the measurement of WMH to total intracranial volume (WMH:intracranial volume). The third substituted total tau and neurofilament light chain (NfL) for the Aβ42/40 ratio. All analysis was performed in Stata 18.0 (StataCorp, College Station, TX) except for the marginal effects figure which was created in Anaconda (Anaconda, Austin, TX). A two‐sided *p*‐value of <0.05 was considered statistically significant.

## 3 RESULTS

**FIGURE 2:**
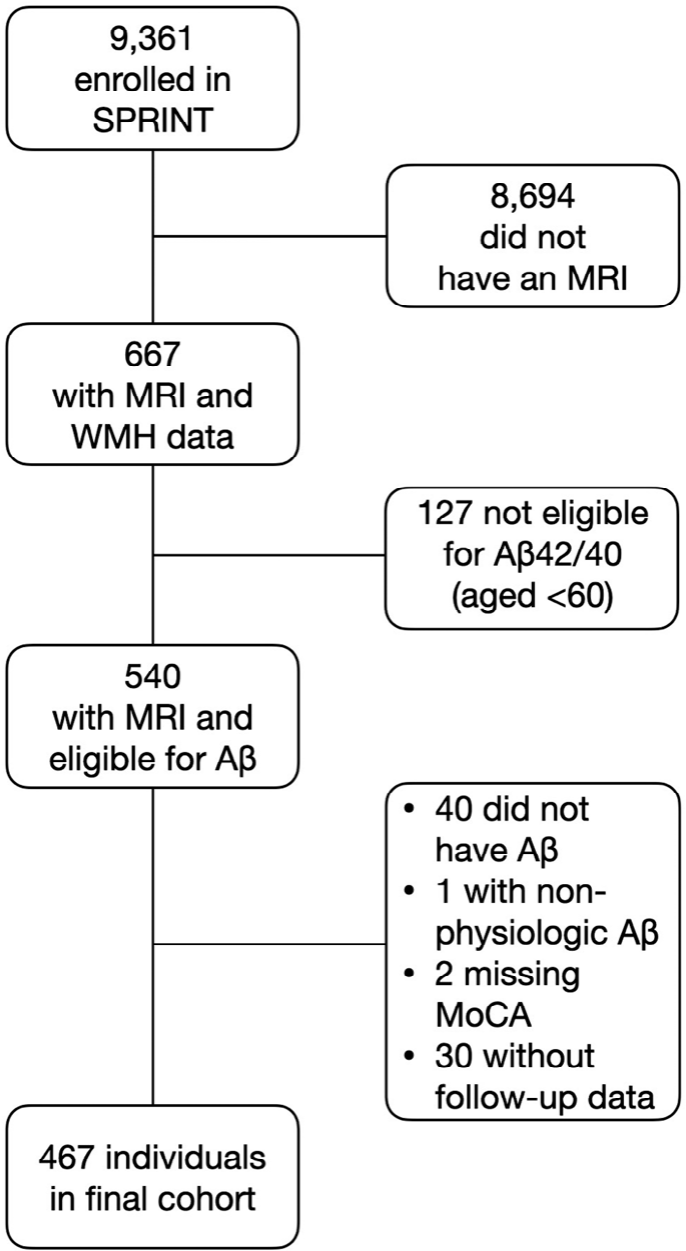
Flowchart of the derivation of the study cohort. Aβ, amyloid beta; MoCA, Montreal Cognitive Assessment; MRI, magnetic resonance imaging; SPRINT, Systolic Blood Pressure Intervention Trial; WMH, white matter hyperintensity.

See Figure 2 for the derivation of our cohort. Of the 9,361 individuals enrolled in SPRINT, 667 had a baseline MRI. Of these, 127 did not have measurement of plasma Aβ42/40 because they were <60 years old. We excluded 40 participants for missing plasma Aβ42/40, 30 for having no follow‐up cognitive outcome data, two for missing baseline MoCA scores, and one for having a non‐physiologic value for Aβ42/40 (>50 standard deviations above mean). The final cohort of 467 individuals had a mean age of 69.7 ± 7.1 years, 41.8% were women, and 31.9% were nonwhite or Hispanic. The differences between included versus excluded SPRINT participants are seen in Table S1. Included individuals were slightly older, more likely to be women, white, and randomized to intensive blood pressure reduction, but less likely to have baseline cardiovascular disease, be smokers, or be uninsured. There were no significant differences in the primary or secondary outcomes between those included versus excluded.

**FIGURE 3:**
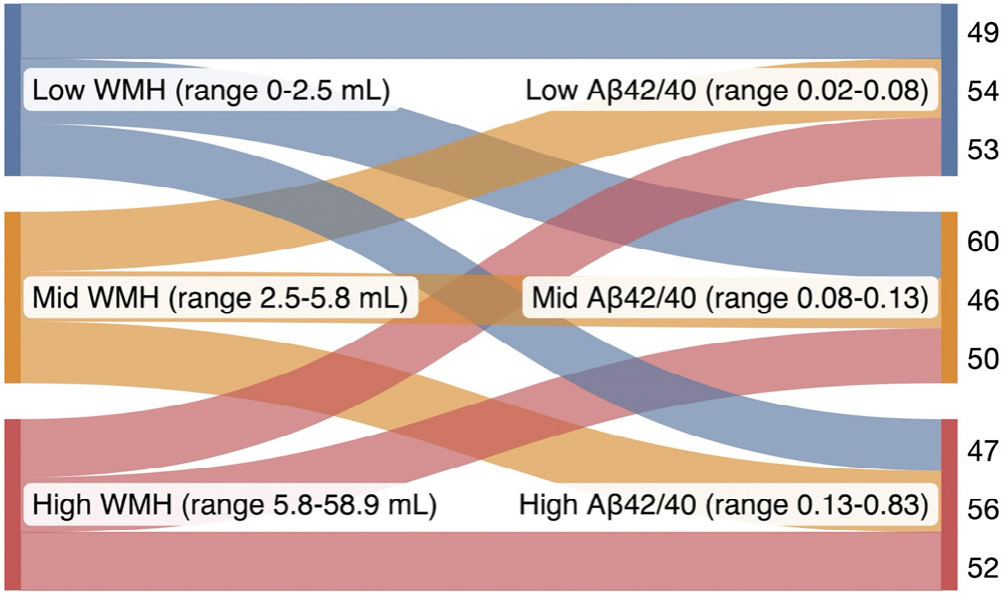
Tertiles of white matter hyperintensity (WMH) in mL and plasma amyloid beta (Aβ)42/40 ratio with ranges reported for each tertile, showing near completely balanced overlap of categories with the number of individuals in each flow shown on the right of the figure.

Comparing the combined low‐risk, Aβ risk, WMH risk, and combined high‐risk categories, there were differences in age and smoking status. Compared to combined low‐risk, participants in the combined high‐risk category were older (75.3 vs 68.1 years) and less likely to be never smokers (34.0% vs 44.0%) (Table 1). There were no significant differences in race/ethnicity, sex, education, physical activity level, or MoCA score across the categories. The exposures of WMH and Aβ42/40 were not correlated (rho = 0.024, *p* = 0.605, Figure 1). After transformation into tertiles, they had a nearly completely balanced overlap of categories, allowing the creation of our exposure categories (Figure 3, which also shows the ranges of WMH in mL and the Aβ42/40 ratio in the tertiles).

**TABLE 1:** Baseline demographics by level of WMH in mL and plasma Aβ42/40 ratio.

	Combined low risk (*n* = 209)	Aβ risk (*n* = 103)	WMH risk (*n* = 102)	Combined high risk (*n* = 53)
Age (mean, SD)	68.1, 6.2	68.7, 6.7	71.1, 7.1	75.3, 7.7
Female sex (%)	38.8%	39.8%	47.1%	47.2%
Race/ethnicity (%)				
White (*n* = 318)	67.0%	72.8%	63.7%	71.7%
Black (*n* = 130)	30.1%	22.3%	32.4%	20.8%
Hispanic (*n* = 14)	2.4%	2.9%	1.9%	7.5%
Other (*n* = 5)	0.5%	1.9%	2.0%	0%
Diabetes (%)	1.9%	2.9%	3.9%	1.9%
Cardiovascular disease (%)	12.9%	16.5%	12.8%	18.9%
Depression (%)	17.7%	16.5%	15.7%	20.8%
Smoking status (%)				
Never (*n* = 217)	44.0%	56.3%	48.0%	34.0%
Prior (*n* = 209)	48.3%	32.0%	42.2%	60.4%
Current (*n* = 41)	7.7%	11.7%	9.8%	5.6%
Current alcohol use (%)	16.3%	13.6%	13.7%	18.9%
Education (%)				
<College (*n* = 264)	55.0%	56.3%	56.8%	62.3%
College (*n* = 72)	15.3%	16.5%	16.7%	13.2%
Graduate school (*n* = 131)	29.7%	27.2%	26.5%	24.5%
Uninsured (%)	8.1%	10.7%	3.9%	3.8%
Activity level				
Inactive (*n* = 183)	37.8%	35.0%	42.2%	47.2%
1–4 d/week (*n* = 224)	46.9%	53.4%	44.1%	49.1%
≥5 d/week (*n* = 60)	15.3%	11.6%	13.7%	3.8%
MoCA (median, IQR)	24, 21–27	24, 22–26	23, 20–26	23, 20–25
Randomized to intensive BP reduction arm (%)	55.0%	59.2%	55.9%	52.8%
Mild cognitive impairment during follow‐up (%)	4.3%	4.9%	10.8%	15.1%
Probable dementia during follow‐up (%)	1.0%	3.9%	1.0%	9.4%
Mild cognitive impairment/dementia during follow‐up (%)	5.3%	7.8%	11.8%	22.6%
All‐cause death during follow‐up (%)	1.0%	3.9%	4.9%	0%
WMH (median, IQR)	2.4, 1.5–3.5	2.7, 1.4–4.2	11.1, 7.1–16.7	11.8, 7.5–19.1
Aβ42/40 (median, IQR)	0.13, 0.10–0.22	0.07, 0.06–0.07	0.13, 0.10–0.25	0.07, 0.06–0.07

Abbreviations: Aβ, amyloid beta; BP, blood pressure; IQR, interquartile range; MoCA, Montreal Cognitive Assessment; WMH, white matter hyperintensity.

**FIGURE 4:**
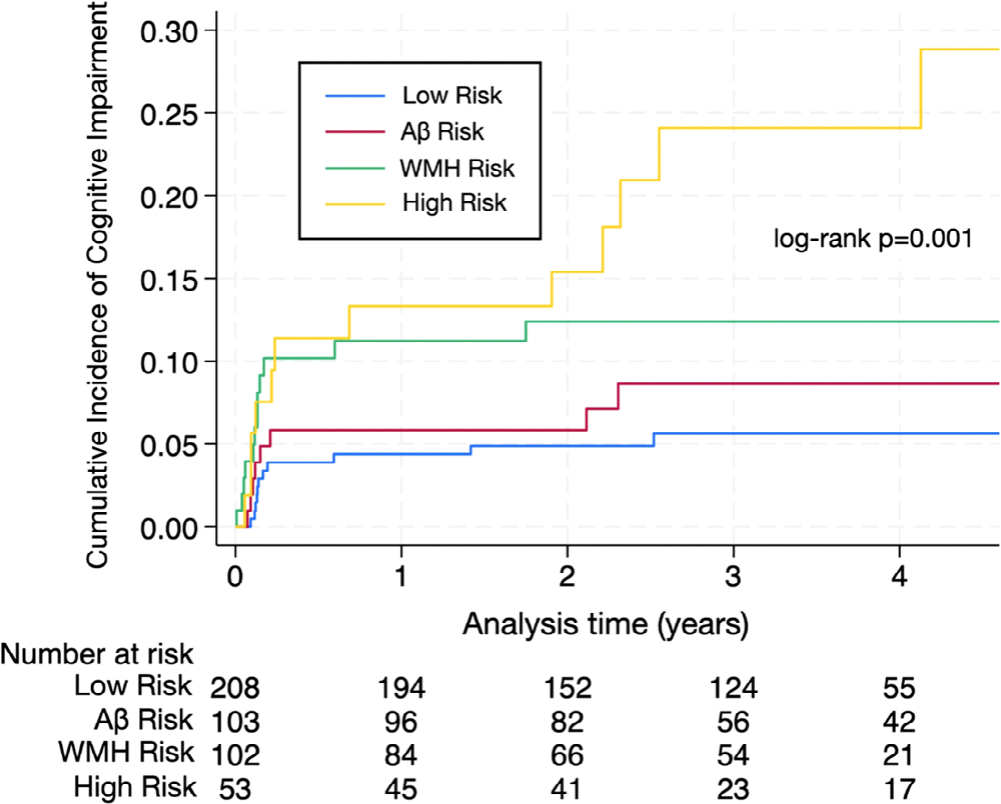
Kaplan–Meier curve for cognitive impairment by baseline category of white matter hyperintensity (WMH) in mL and plasma amyloid beta (Aβ)42/40 ratio. To assist in visual interpretation the index time was set at 1.9 years from enrollment when the follow‐up cognitive assessment started.

Of the 467 participants, 43 (9.2%) developed cognitive impairment during a mean 4.8 ± 1.4 years of follow‐up. Of these 43 primary outcome events, 12 were dementia and 31 were MCI. The proportion of individuals with cognitive impairment was 5.3%, 7.8%, 11.8% and 22.6%, respectively, across the combined low‐risk, Aβ risk, WMH risk, and combined high‐risk categories (*p* < 0.001). The Kaplan–Meier curve for a cumulative incidence of cognitive impairment by exposure category is shown in Figure 4. Compared to the combined low‐risk category, those in the combined high‐risk category had an unadjusted hazard ratio (HR) for cognitive impairment of 4.45 (95% confidence interval [CI], 1.96 to 10.1) and 4.12 (95% CI, 1.71 to 9.94) after adjustment (Table 2). Participants in the Aβ and WMH risk categories had adjusted HRs of 1.49 (95% CI, 0.59 to 3.76) and 2.20 (95% CI, 0.96 to 5.05), respectively.

**TABLE 2:** Cognitive impairment and dementia outcomes by baseline category of WMH and plasma Aβ42/40 ratio.

	Proportion with event	Incidence rate (95% CI) per 1,000 person‐years	Unadjusted hazard ratio (95% CI)	*p*‐value	Adjusted hazard ratio (95% CI)^*^	*p*‐value
Cognitive impairment (composite outcome including probable dementia and MCI)						
Combined low risk (*n* = 209)	5.3%	10.8 (6.0–19.4)	Ref	Ref	Ref	Ref
Aβ risk (*n* = 103)	7.8%	15.5 (7.7–30.9)	1.46 (0.59–3.63)	0.417	1.49 (0.59–3.76)	0.401
WMH risk (*n* = 102)	11.8%	25.8 (14.7–45.5)	2.45 (1.08–5.56)	0.032	2.20 (0.96–5.05)	0.063
Combined high risk (*n* = 53)	22.6%	48.3 (27.4–85.0)	4.45 (1.96–10.1)	<0.001	4.12 (1.71–9.94)	0.002
Dementia						
Combined low risk (*n* = 209)	1.0%	1.9 (0.5–7.6)	Ref	Ref	Ref	Ref
Aβ risk (*n* = 103)	3.9%	7.6 (2.8–20.1)	3.98 (0.73–21.8)	0.111	5.44 (0.97–30.6)	0.054
WMH risk (*n* = 102)	1.0%	2.0 (0.3–14.3)	1.08 (0.10–11.9)	0.951	1.02 (0.09–11.4)	0.986
Combined high risk (*n* = 53)	9.4%	18.8 (7.8–45.1)	9.68 (1.87–50.0)	0.007	7.48 (1.33–42.0)	0.022

Abbreviations: Aβ, amyloid beta; CI, confidence interval; MCI, mild cognitive impairment; WMH, white matter hyperintensity.

* When fit to cognitive impairment, model is adjusted for baseline age, sex, race, education, physical activity level, alcohol use, and randomization arm. When fit to dementia, model is adjusted for baseline age, sex, race.

In the first sensitivity analysis, we used a Fine‐Gray model to treat all‐cause death as a competing event and the adjusted HR for cognitive impairment for the combined high‐risk category was 4.17 (95% CI, 1.86 to .38). In the second sensitivity analysis, where WMH was standardized to total intracranial volume, the adjusted HR for cognitive impairment for the combined high‐risk category was 3.65 (95% CI, 1.49 to 8.95). In the third sensitivity analysis, we used total tau and NfL instead of Aβ. We did not find that total tau was predictive of cognitive impairment or additive when combined with WMH. NfL was predictive of cognitive impairment when combined with WMH, but with a smaller effect size than Aβ (HR 3.13; 95% CI, 1.23 to 7.96) (Table 3).

**TABLE 3:** Cognitive impairment outcomes by baseline category of WMH and plasma total tau and neurofilament light chain (NfL).

	Proportion with event	Adjusted hazard ratio (95% CI)^*^	*p*‐value
Total Tau (*n* = 466)			
Combined low risk (*n* = 225)	5.8%	Ref	Ref
Tau risk (*n* = 87)	6.9%	1.18 (0.44–3.16)	0.746
WMH risk (*n* = 86)	20.9%	3.47 (1.66–7.29)	0.001
Combined high risk (*n* = 68)	8.8%	1.39 (0.51–3.80)	0.516
Neurofilament light chain (NfL) (*n* = 457)			
Combined low risk (*n* = 219)	5.0%	Ref	Ref
NfL risk (*n* = 85)	8.2%	1.54 (0.58–4.14)	0.389
WMH risk (*n* = 86)	15.1%	2.73 (1.20–6.22)	0.017
Combined high risk (*n* = 67)	16.4%	3.13 (1.23–7.96)	0.016

Abbreviation: CI, confidence interval; WMH, white matter hyperintensity.

* Model is adjusted for baseline age, sex, race, education, physical activity level, alcohol use, and randomization arm.

The secondary outcome of dementia occurred in 1.0%, 3.9%, 1.0%, and 9.4% of individuals, respectively, across the combined low‐risk, Aβ risk, WMH risk, and combined high‐risk categories (*p* = 0.003). Compared to the combined low‐risk category, those in the combined high‐risk category had an unadjusted HR for dementia of 9.68 (95% CI, 1.87 to 50.0), which was 7.48 (95% CI, 1.33 to 42.0) after adjustment for age, sex, and race (Table 2).

**FIGURE 5:**
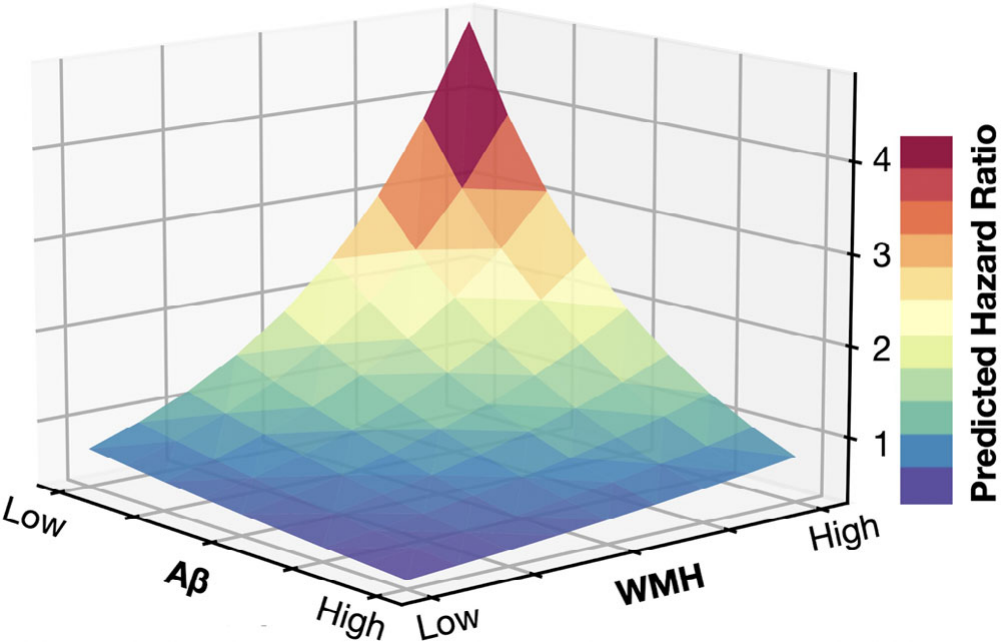
White matter hyperintensity (WMH) volume in mL and amyloid beta (Aβ)42/40 ratio as continuous variables after cube‐root transformation, introduced as an interaction term in the Cox model fit to cognitive impairment. Marginal effects of their centered mean plus or minus two standard deviations are shown.

For analyses with the primary outcome of cognitive impairment, the interaction terms between the risk categories and age, sex, race/ethnicity, and baseline MoCA were not significant. In the exploratory analysis where Aβ and WMH are cube‐root transformed and treated as continuous variables, the 3D plot showing the HR for cognitive impairment is shown in Figure 5. In this analysis, the risk of cognitive impairment increases linearly from the centered mean across two standard deviations of both Aβ and WMH.

The interaction term of binary WMH*Aβ42/40 was not significant (HR 1.52; 95% CI, 0.41 to 5.67; *p* = 0.533). The RERI was 1.43 (95% CI, −1.67 to 4.54), the attributable proportion was 0.35 (95% CI, −0.24 to 0.94), and the synergy index was 1.85 (95% CI, 0.47 to 7.37), confirming that the exposures are additive, but not multiplicative or superadditive. However, the wide 95% CI of the interaction term and the RERI statistic precludes definitive rejection of a multiplicative or superadditive relationship between WMH and Aβ42/40.

## 4 DISCUSSION

In a population of hypertensive adults aged ≥60, we found that having both high‐risk WMH on MRI and Aβ on plasma measurement correlates with a fourfold increase in the odds of developing cognitive impairment over almost 5 years of follow‐up. WMH and Aβ were not correlated so we did not perform mediation analysis. We also found that WMH and Aβ were not multiplicative, but the combined fourfold increase in risk exceeds that of having increased levels of either on their own. These findings and the RERI of 2.11 confirm that WMH and Aβ are additive, which is consistent with most prior research on this topic showing independent or additive effects of WMH and Aβ on cognition.^26^, ^27^, ^29^, ^42^ However, other research has suggested a multiplicative effect.^28^, ^43^ Differences in the cohorts of these studies could account for the inconsistent effects. Ultimately, a clinical trial that treats both amyloid and vascular risk factors like hypertension in a mixed etiology dementia population would be needed to understand the actual relationship.

In broad terms, dementia biomarker research has predominantly included non‐Hispanic white participants and/or involved participants with potential AD.^9^, ^10^, ^11^, ^22^ Our findings reinforce the paradigm of mixed contributions to all‐cause cognitive impairment in a diverse population with the vascular risk factor of hypertension. This broad inclusivity strengthens the generalizability of the results and underscores the importance of considering both WMH and Aβ in cognitive risk assessments across varied ethnic and racial backgrounds. Black and Hispanic patients have the highest prevalence of dementia in the United States.^44^ Furthermore, in patients with dementia, WMH is more common in Hispanic and non‐Hispanic black individuals.^45^

SPRINT showed that intensive blood pressure reduction (<120 mm Hg), compared to a target of <140 mm Hg, significantly reduced the rate of probable dementia/MCI (HR 0.85; 95% CI, 0.74 to 0.97) but not of probable dementia alone (HR 0.83; 95% CI, 0.67 to 1.04).^30^ Intensive blood pressure reduction in SPRINT reduced the progression of WMH,^33^ but not the level of plasma Aβ.^34^ This finding supports the central role of hypertension in the development and progressive accumulation of WMH and the independence of Aβ pathology. However, elderly individuals with hypertension can also have Aβ deposition. Much like WMH, Aβ burden increases with age. By age 70 (the mean age in this cohort) over half of individuals without dementia will have evidence of either WMH or Aβ on brain scans.^12^, ^13^, ^14^, ^46^, ^47^ The “independent but additive” narrative^27^, ^28^, ^29^ is consistent with our finding that the highest risk of cognitive impairment is seen in individuals who have both pathologies.

Monoclonal antibody therapies targeting Aβ have been approved by the FDA for preventing progression of AD, but the anti‐amyloid drugs have a more impressive effect on Aβ clearance from the brain than cognitive outcomes.^48^, ^49^, ^50^ There are multiple hypotheses regarding the origin of the residual cognitive decline with anti‐amyloid therapies.^51^, ^52^, ^53^ Nonetheless, these findings parallel the effect of intensive blood pressure reduction in SPRINT, which led to a significant reduction of WMH progression but not in the development of dementia.^30^, ^33^ SPRINT did not treat Aβ pathology in eligible individuals and the recent trials testing monoclonal antibody therapies targeting Aβ (eg, Clarity AD, TRAILBLAZER, or A4) did not treat hypertension or other vascular risk factors when present.^48^, ^49^, ^50^, ^54^, ^55^, ^56^ Clinical trials of treatments to prevent dementia have largely been siloed in separate vascular or amyloid spheres. Recognizing this, the National Institutes of Health (NIH) is currently accepting applications for a phase 3 clinical trial to test FDA‐approved monoclonal antibody therapies targeting amyloid in diverse mixed dementia populations.^57^

### 4.1 Limitations

Despite the strengths of this well‐phenotyped cohort with almost 5 years of follow‐up, rigorous prospective adjudication of cognitive outcomes, and over 30% nonwhite participants, there are major limitations that should be acknowledged. The post hoc hypothesis‐generating nature of our study precludes establishing causality. In addition, the small size of the cohort and small number of outcomes leads to imprecision and low power. The exclusion of participants due to missing data also introduces selection bias and limits the generalizability of the study's findings. The burden of WMH in SPRINT MIND participants in the top tertile of WMH was moderate in most and severe in a minority, based on ordinal scales such as the Fazekas score.^58^ This also limits generalizability of our study.

The analyzer and assay used to measure Aβ are not the most current versions, which could have introduced measurement errors. In addition, plasma testing of Aβ is less specific than testing of cerebrospinal fluid or obtaining PET scans. We could not account for potential confounders like genetic predisposition (apolipoprotein E [*APOE*]), dietary habits, or other comorbidities, which could play roles in both WMH and Aβ accumulation and associated risk. We were not able to examine the potential contribution of other downstream biomarkers of vascular cognitive impairment including lacunar strokes or cerebral microhemorrhages, which may have been informative in this cohort but were not adjudicated on the SPRINT MIND MRI. Lastly, the categorization into risk groups, although useful for stratified analysis, may oversimplify this complex continuum of pathology.

## CONFLICT OF INTEREST STATEMENT

Dr. de Havenon has received consultant fees from Novo Nordisk, royalty fees from UpToDate, and has equity in TitinKM and Certus. Dr. Sheth reports compensation from Sense and Zoll, for data and safety monitoring services; compensation from CereVasc, CSL Behring, Rhaeos, and Astrocyte for consultant services; a patent for Stroke wearables licensed to Alva Health. Dr. Brickman serves as a scientific advisor/consultant to Cognito Therapeutics, Cognition Therapeutics, and Cogstate. He serves on data safety and monitoring boards for Albert Einstein College of Medicine and University of Illinois. Dr. Brickman has a patent for white matter hyperintensity quantification (patent # 9867566) and a patent pending for microbleed detection (publication #20230298170). Dr. Sharma has a provisional patent for an ischemic stroke etiology classification algorithm (No.63/505,006). Dr. Schneider reports serving as an Associate Editor for the journal *Neurology* from the American Academy of Neurology. Dr. Pajewski reports serving as an Assistant Editor for the *Journal of the American Geriatrics Society* and as a Statistical Editor for the journal *Hypertension* from the American Heart Association. He also serves on data and safety monitoring boards for Atrium Health, Brigham and Women's Hospital, Indiana University School of Medicine, Medical University of South Carolina, University of California–San Francisco, and the University of Texas–San Antonio. Vivian Li reports no disclosures. Lauren Littig reports no disclosures. Dr. Gottesman reports no disclosures. Dr. Gottesman reports no disclosures. Dr. Williamson reports no disclosures. Dr. Rost reports no disclosures. Dr. Stulberg reports no disclosures. Dr. Falcone reports no disclosures. Dr. Prabhakaran reports no disclosures. Author disclosures are available in the supporting information.

## CONSENT STATEMENT

All human subjects in the SPRINT and SPRINT MIND trials provided informed consent to participate in the clinical trial.

## Supporting information

Supporting Information

Supporting Information
