# Supplementary material for: White matter hyperintensity on MRI and plasma Aβ42/40 ratio additively increase the risk of cognitive impairment in hypertensive adults
Source: Alzheimers Dement. 2024 Sep 4;20(10):6810–9. doi: 10.1002/alz.14126 (PMC11485393; doi:10.1002/alz.14126)
Supplement: Supplementary file 1 — Supporting Information [file ALZ-20-6810-s002.docx]

**SUPPLEMENTARY FILES**

**Supplementary Table 1.** Baseline demographics by inclusion versus exclusion.

|  | **Included**  (n=467) | **Excluded**  (n=8,894) | **P value*** |
| --- | --- | --- | --- |
| Age (mean, SD) | 69.7, 7.1 | 67.8, 9.5 | <0.001 |
| Female (%) | 41.8% | 35.3% | 0.004 |
| Race/ethnicity (%)  White (n=5,400)  Black (n=2,809)  Hispanic (n=985)  Other (n=167) | 68.1%  27.8%  3.0%  1.1% | 57.1%  30.1%  10.9%  1.8% | <0.001 |
| Diabetes (%) | 2.6% | 1.6% | 0.103 |
| Cardiovascular disease (%) | 14.4% | 20.4% | 0.002 |
| Smoking status (%)  Never (n=4,121)  Prior (n=4,000)  Current (n=1,240) | 46.5%  44.7%  8.9% | 43.9%  42.6%  13.5% | 0.014 |
| Current alcohol use (%) | 15.4% | 14.4% | 0.549 |
| Education (%)  <College (n=5,709)  College (n=1,353)  Graduate (n=2,283) | 56.5%  15.4%  28.1% | 61.3%  14.4%  24.3% | 0.100 |
| Uninsured (%) | 7.3% | 10.6% | 0.024 |
| Activity level  Inactive (n=4,032)  1-4d/week (n=4,029)  ≥5d/week (n=1,253) | 39.1%  48.0%  12.9% | 43.5%  43.0%  13.5% | 0.102 |
| MOCA  (median, IQR) | 24, 21-26 | 23, 20-26 | 0.025 |
| Randomized to intensive BP reduction arm (%) | 55.9% | 49.7% | 0.009 |
| Mild cognitive impairment during follow-up (%) (n=8,563) | 7.1% | 7.5% | 0.730 |
| Probable dementia during follow-up (%) (n=8,563) | 2.6% | 3.9% | 0.154 |
| Mild cognitive impairment/dementia  during follow-up (%) (n=8,563) | 9.2% | 10.2% | 0.478 |

* Intergroup differences tested with chi-squared, Student’s t-test, and Wilcoxon ranksum for binary, continuous, and nominal/ordinal variables, respectively.
